# Excision of Oxidatively Generated Guanine Lesions by Competitive DNA Repair Pathways

**DOI:** 10.3390/ijms22052698

**Published:** 2021-03-07

**Authors:** Vladimir Shafirovich, Nicholas E. Geacintov

**Affiliations:** Chemistry Department, New York University, New York, NY 10003-5180, USA; nicholas.geacintov@nyu.edu

**Keywords:** DNA damage, base excision repair, nucleotide excision repair, oxidative stress, reactive oxygen species, guanine oxidation

## Abstract

The base and nucleotide excision repair pathways (BER and NER, respectively) are two major mechanisms that remove DNA lesions formed by the reactions of genotoxic intermediates with cellular DNA. It is generally believed that small non-bulky oxidatively generated DNA base modifications are removed by BER pathways, whereas DNA helix-distorting bulky lesions derived from the attack of chemical carcinogens or UV irradiation are repaired by the NER machinery. However, existing and growing experimental evidence indicates that oxidatively generated DNA lesions can be repaired by competitive BER and NER pathways in human cell extracts and intact human cells. Here, we focus on the interplay and competition of BER and NER pathways in excising oxidatively generated guanine lesions site-specifically positioned in plasmid DNA templates constructed by a gapped-vector technology. These experiments demonstrate a significant enhancement of the NER yields in covalently closed circular DNA plasmids (relative to the same, but linearized form of the same plasmid) harboring certain oxidatively generated guanine lesions. The interplay between the BER and NER pathways that remove oxidatively generated guanine lesions are reviewed and discussed in terms of competitive binding of the BER proteins and the DNA damage-sensing NER factor XPC-RAD23B to these lesions.

## 1. Introduction

Endogenous reactive oxygen species, free radicals and electrophiles generated by UV light, ionizing radiation and environmental pollutants, are known to induce permanent DNA damage and harmful epigenetic changes [1,2,3]. It has been estimated that up to 10^5^ spontaneous or induced DNA lesions are formed per cell every day [4]. Typical forms of nucleotide modifications, include DNA cross-links, strand breaks, and a variety of oxidatively generated DNA lesions that enhance the rates of mutations and genomic instability [5,6]. In healthy tissues, genomic stability is maintained by the DNA repair machinery that removes DNA lesions in an efficient and timely manner [7]. Among the multiple forms of cellular repair, the base excision repair (BER) pathway generally removes non-bulky oxidatively generated DNA lesions [8].

The mechanisms of BER are highly conserved from bacteria to humans and involve the excision of single DNA lesions [9], while DNA helix-distorting bulky lesions are recognized, excised and repaired by the nucleotide excision repair (NER) mechanism [10]. The structural distortions caused by DNA lesions are recognized by the DNA damage-sensing NER factor XPC-RAD23B (abbreviated as XPC) [11]. The initial XPC binding step constitutes the recognition stage that is followed by the sequential binding of the ten-protein factor TFIIH and XPA, XPF and XPG proteins to the site of the lesion. The hallmark of the NER excision mechanism is the appearance of the characteristic ~24–30 nucleotide (nt) dual incision products that contain the lesion [10]. Deficiency in XPC levels is associated not only with decreased rates of repair of certain oxidatively generated DNA lesions, but also to compromised redox homeostasis and loss of cell cycle control [12,13].

Growing evidence suggests that the BER and NER pathways can compete with one another in removing oxidatively generated lesions from double-stranded DNA [14,15,16,17]. In this contribution, we summarize more recent experimental results that provide further insights into the competition between the BER and NER pathways in excising hydantoin lesions that are generated by the oxidation of guanine [14,18,19,20,21]. A significant enhancement (by factor of 5–6) of the NER dual incision yields of hydantoin lesions embedded in covalently closed circular DNA plasmids (cccDNA) than in the linearized form of the same plasmid DNA (linDNA) has been observed [21,22]. By contrast, the BER yields in cccDNA and linDNA did not vary by more than 20–40%, depending on the guanine lesion. These surprising differences in NER and BER activities have been attributed to the lack of termini in covalently closed circular DNA, that enhance the search dynamics of the NER DNA damage sensor XPC in circular DNA plasmid molecules.

## 2. Guanine Lesions Generated by Electron Abstraction and Free Radical Oxidation Pathways

Guanine, the most easily oxidizable nucleic acid base [23], is a primary target of oxidizing agents [24]. Among the four canonical DNA bases, guanine has the lowest redox potential (*E*_7_ = 1.26 V vs. NHE [23]), and is readily oxidized by carbonate radical anions (CO_3_^•-^), a mild oxidizing agent (*E*^0^ = 1.59 V vs. NHE [25]). The carbonate radical anion is generated in cellular environment by the decomposition of nitrosoperoxycarbonate (ONOOCO_2_) [2]. Oxidation of other nucleobases (A, C, and T) [26,27,28] requires significantly stronger oxidants, such as the sulfate radical, SO_4_^•−^ (*E*^0^ = 2.43 V vs. NHE [25].

Guanine oxidation is typically initiated either by one-electron abstraction or by hydroxyl radical addition reactions (Figure 1A) [15,26].

The free radical intermediates, the guanine radical cation (G^•+^) and the neutral guanine radical [G(-H)^•^] are highly reactive, and are rapidly hydrated via the addition of H_2_O molecules to either the C5 or C8 position of guanine [34,35]. The 8-HO-G^•^ and 5-HO-G^•^ radicals formed, which are identical to the radical adducts derived from the addition of hydroxyl radicals to the C8 and C5 positions, are reducing agents. These intermediates are rapidly oxidized by O_2_ or other relatively weak oxidants, to form two stable end-products of two-electron oxidation, 8-oxo-7,8-dehydroguanine (8-oxoG) and 5-carboxamido-5-formamido-2-iminohydantoin (2Ih) [34,35,36,37,38]. The latter exists in the form of two diastereomers, *R*-2Ih and *S*-2Ih, which are chemically stable and can be individually isolated by HPLC methods [34,39]. Alshykhly et al. [30] reported that both 2Ih diastereomers are typical BER substrates and can be removed from damaged DNA by the glycosylases NEIL1 and Fpg.

It has been demonstrated that unrepaired, oxidatively generated DNA lesions are associated with germline mutations in tumor suppressor genes and proto-oncogenes that lead to adenoma-colorectal cancers [40]. The widely studied 8-oxoG product is one of the most abundant and best characterized oxidatively generated DNA lesion, and is a classical BER substrate that is formed in cellular environments under conditions of oxidative stress [21,41,42]. The 8-oxoG lesions are genotoxic, and failure to remove 8-oxoG before replication occurs, results in the formation of G:C→T:A transversion mutations [43]. Furthermore, 8-oxoG is more easily oxidized than the parent guanine [44], and reacts with diverse oxyl radicals (CO_3_^•−^, ^•^NO_2_, SO_4_^•−^, RO^•^) [34,45,46,47,48,49,50], and peroxynitrite [51,52] to yield the stable end-products of four-electron oxidation of guanine, spiroiminodihydantoin (Sp) and 5-guanidinohydantoin (Gh) [53,54,55,56,57,58,59]. Due to the presence of chiral carbon atoms, Sp and Gh exist in two diastereoisomeric *S* and *R* forms. The Sp-modified oligodeoxynucleotides can be separated and purified by anion-exchange HPLC methods [60,61]. In contrast, the Gh diastereomers are easily interconvertible, and can isomerize to iminoallantoin (Ia) (Figure 1A) [54]. In DNA, isomerization of Gh to Ia occurs in basic solutions (pH > 8.2) [62]. The Sp and Gh lesions have been detected in mice with infection-induced colitis, at concentration levels of about one percent, relative to the more abundant 8-oxoG levels [63]. The hydantoin lesions are at least one order of magnitude more mutagenic than the parent 8-oxoG (mainly via G→C and G→T transversion mutations) [64]). The hydantoin lesions are efficiently repaired by BER enzymes that include the prokaryotic *E. coli* Fpg [65] and Nei [66], the mammalian NEIL1 and NEIL2 [67], NEIL3 [68,69,70,71,72], and the human NEIL1 [31,73] and NEIL3 [72] DNA glycosylases. These lesions are substrates of prokaryotic BER and NER mechanisms [74], in cell extracts from HeLa cells and human fibroblasts [14,21], as well as in intact HeLa cells and human fibroblasts [19]. As a positive control of NER activity, we employed the bulky 10*R*-(+)-*cis-anti*-B[*a*]PDE-*N*^2^-dG adduct (abbreviated as B[*a*]P-dG), an excellent substrate of the human NER pathway [14,19,22,32,33].

## 3. Construction of Plasmid Substrates Harboring Single Guanine Lesions by a Gapped-Vector Technology

**Figure 2:**
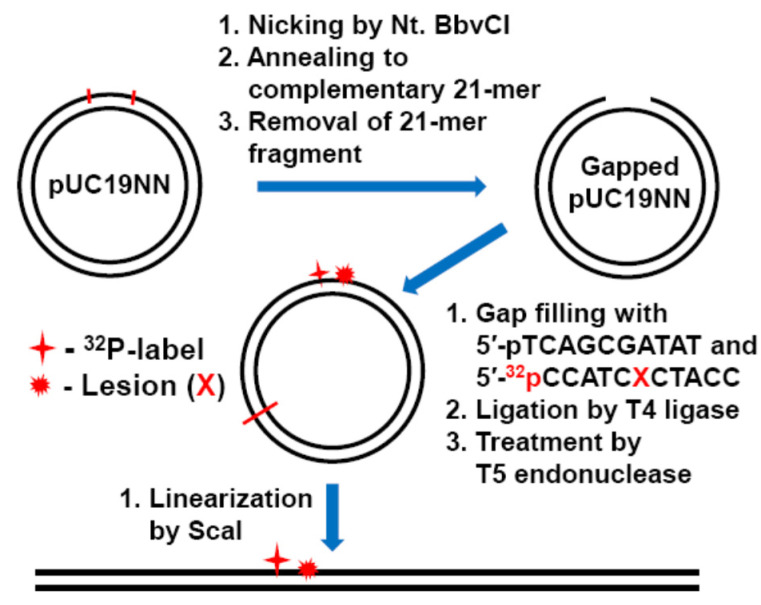
Covalently closed circular and linearized plasmid (2686 bp) containing site-specifically positioned guanine lesions (X) and a radioactive ^32^P-internal label. The circular plasmid substrates were generated by gapped-vector technology from a pUC19NN plasmid containing two Nt. BbvCI restriction sites [21,22]. The linearized plasmid substrate with the lesion X positioned at the 945th nucleotide counted from the 5′-end, was prepared by the selective cleavage of the circular plasmid with a unique ScaI restriction enzyme that generates blunt end cleavage products.

Recently we demonstrated that the interplay of BER and NER pathways in covalently closed circular plasmids dramatically differs from that in the same, but linearized plasmids [21,22]. A gapped-vector technology [75,76,77,78,79,80] was employed for the site-specific insertion of single guanine lesions into pUC19NN plasmid molecules (contour length of 2686 bp). The parent pUC19NN plasmid was cloned from the well-known pUC19 plasmid by inserting a 32-mer 2′-deoxyoligonucleotide fragment containing two Nt. BbvCI restriction sites separated by 21 nucleotides (Figure 2).

The gapped plasmid derived from the nicking of pUC19NN with the Nt. BbvCI restriction enzyme, was filled with the 5′-phosphorylated oligonucleotides 5′-pTCAGCGATAT and ^32^P-endlabeled 5′-^32^pCCATCXCTACC (where the lesion X = Gh, *S*-Sp, 8-oxoG, or *cis*-B[*a*]P-dG), and was ligated to the plasmid by T4 ligase. The reaction products were treated with T5 exonuclease to digest any linear and nicked plasmids [79,80,81]. The cleavage of the covalently closed circular plasmid substrates by the unique restriction enzyme ScaI generates linearized plasmids with blunt ends with a guanine lesions (X) at the 945th nucleotide counted from the 5′-end.

## 4. Monitoring Competing BER and NER Pathways with Single DNA Lesions Embedded in Plasmids

**Figure 3:**
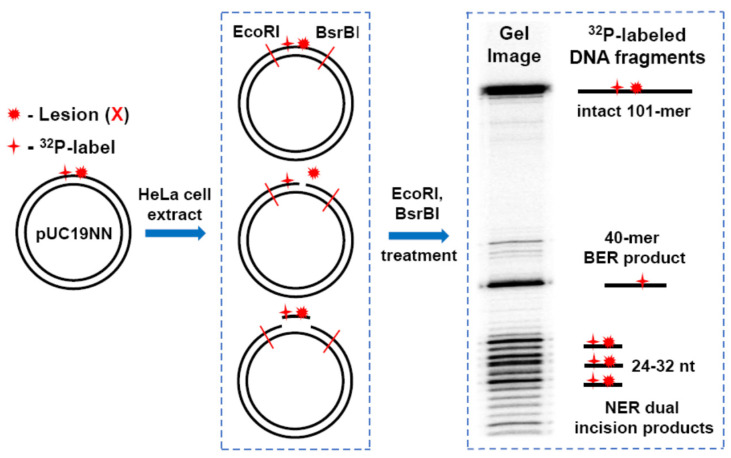
Schematic summary of the analysis of products derived from the incisions of covalently closed circular plasmids by BER and NER mechanisms after incubation in human cell extracts [21,22].

The formation of NER and BER excision products was monitored by ^32^P-internally labelled plasmid substrates harboring single lesions, by high resolution denaturing polyacrylamide gel electrophoresis methods [21,22]. After incubation of the plasmid substrates in cell extracts, the DNA samples were isolated and treated with EcoRI and BsrBI restriction enzymes to excise ^32^P-labeled 40-mer fragments, the products of successful BER activity, or the unincised and intact 101-mer fragments as shown in Figure 3.

Successful NER activity generates the dual incision products ~24–32 nucleotides in lengths containing the ^32^P-label, as well as the lesion [82,83]. A similar approach was employed to determine the yields of BER and NER excision products using linDNA containing single Sp and Gh lesions [21,22].

## 5. Remarkable Enhancement of NER of Guanine Lesions in Covalently Closed Circular Plasmids Relative to the Same, But Linearized Plasmids

We recently discovered that the NER dual incision yields are enhanced by factor of 5–6 when guanine lesions are embedded in covalently closed circular pUC19NN plasmids rather than in the same, but linearized plasmid containing single Sp, Gh lesions or (+)-*cis*-B[*a*]P-dG adducts by treatment of the circular pUC19NN plasmid with the restriction enzyme ScaI (Figure 2) [21,22]. In these experiments, we used the hydantoin lesions (Gh and *S*-Sp), which are substrates of both BER and NER pathways, and employing 8-oxoG as a positive controls of the BER and NER pathways, respectively.

The denaturing polyacrylamide gel electrophoresis clearly show that incubation of the *S*-Sp-plasmids in HeLa cell extracts followed by treatment with EcoRI and BsrBI restriction enzymes generates three groups of ^32^P-labeled DNA fragments: (1) the characteristic ladders of NER dual incision products of ~20–32 nucleotides in lengths, (2) the 40-mer fragments generated by BER activity, and (3) the 101-mer fragment excised by the restriction enzymes from the unincised and intact plasmids (Figure 3) [21]. A similar remarkable enhancement of the NER dual incision yields was also evident in the case of Gh lesions. The 8-oxoG lesions in the same plasmids are exclusively repaired by the BER pathway [21]. In turn, the (+)-*cis*-B[*a*]P-dG adduct is removed by the NER mechanism only, as expected [22].

**Figure 4:**
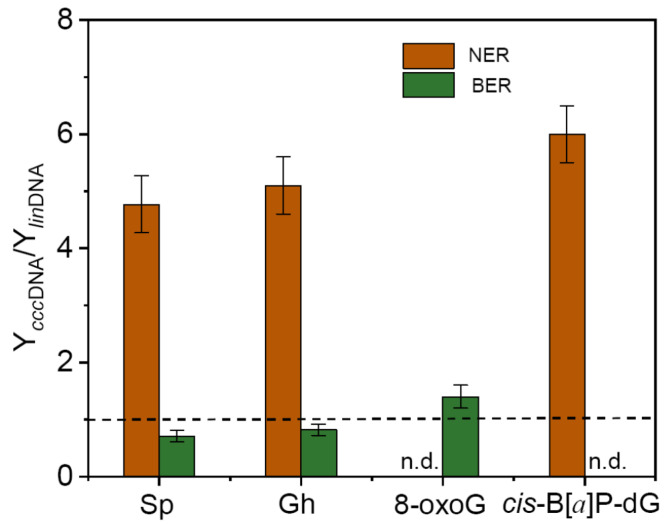
Impact of plasmid linearization on the ratios of the relative yields of BER and NER incision products (Y*_ccc_*_DNA_/Y*_lin_*_DNA_) after incubation of ^32^P-internally labelled plasmids harboring *S*-Sp, Gh, 8-oxoG, and (+)-*cis*-B[*a*]P-dG lesions in HeLa cell extracts. n.d.–not detected. (Data from Kolbanovskiy et al. *Biochemistry*, **2020**, *59*, 2842–2848 [21], and *Chem. Res. Toxicol*., **2021**, *34*, 154–160 [22]). n.d.–not detected.

The effects of plasmid linearization on the ratio of the relative yields of BER and NER incision products (Y*_ccc_*_DNA_/Y*_lin_*_DNA_) [21,22] are summarized in Figure 4.

This figure shows that the (Y*_ccc_*_DNA_/Y*_lin_*_DNA_) ratios are 4.8 ± 0.5 (Sp), 5.1 ± 0.5 (Gh) [21], and 6.0 ± 0.5 (B[*a*]P-dG) [20], thus indicating that the NER activities of both hydantoin lesions and B[*a*]P-dG adduct in circular plasmids are *greater* than in linearized plasmids by a factor of ~5–6. However, the relative BER yields of the Sp and Gh lesions are reduced by ~20–30% in circular plasmids. This decrease in BER activities of Sp and Gh in circular plasmids might be due to the competition between XPC and the BER enzyme for binding to the Sp or Gh lesions [20] since the NER pathway is highly favored in circular DNA molecules. However, a somewhat greater BER activity by ~30–40% in cccDNA is observed in the case of 8-oxo-G in circular plasmids. The remarkable enhancement in NER yields in circular relative to linearized plasmid DNA molecules, suggests the hypothesis that this enhancement is associated with an effectively greater level of bound XPC molecules in circular DNA than in the linearized form. Since the contour lengths and base sequence context are identical in both cases, the smaller NER yield in the linear plasmid molecules is attributed to the two DNA termini that are absent in circular DNA. Single molecule experiments have shown recently that XPC diffuses along linear DNA molecules by a combined sliding and hopping mechanism [84]. The probability of sliding or hopping off the DNA molecule at the ends in linearized DNA molecules is enhanced, thus diminishing the probability that the same XPC molecule will locate the DNA lesion [22]. Indeed, Mason et al. [85] demonstrated earlier that NER excision efficiencies are significantly enhanced in human cell extracts in 149-mer DNA duplexes when the ends are blocked by streptavidin-biotin complexes. Thus, the XPC molecules were prevented from dissociating at both ends of the DNA molecules thus enhancing the observed NER yields. End-effects may play an important role in chromatin remodeling, an important factor in intracellular DNA repair [86,87,88,89]. Whitehouse et al. demonstrated that the ATP-dependent displacement of histone octamer cores induced by SWI/SNF chromatin remodeling complexes was blocked by streptavidin-coated magnetic beads [90].

## 6. Competition of BER and NER Pathways in Repair of Oxidatively Generated Guanine Lesions

The interplay of BER and NER in the repair of hydantoin lesions can be explained by a competitive binding of BER and NER proteins to the Sp and Gh lesions [15]. In principle, the ratios of incision products generated at a given concentration of DNA substrates could be limited by the relative concentrations of one or the other kind of DNA repair protein. Such effects could manifest themselves at different DNA substrate concentrations at constant BER and NER protein concentrations. Indeed, we have recently found that a five-fold rise of DNA concentrations from 0.2 nM to 1 nM induces a decrease in the NER/BER yield ratios from ~4.3 to ~2.7 and ~2.0 to ~1.2 in the case of Sp- and Gh-plasmids, respectively [21]. These results indicate that the observed small decreases in NER/BER ratios as a function of DNA substrate concentration, are due to limiting concentrations of NER proteins in cell extracts.

The enhancement of BER yields induced by the addition of DNA glycosylase NEIL1 is correlated with the suppression of the yields of NER dual incision products in cell extracts; this observation indicates that the NER/BER ratios are determined by a competition between NEIL1 and the initial NER DNA lesion recognition factor XPC [14]. Recent experiments with purified human NEIL1 and XPC proteins provide direct support for the competitive binding model of these proteins to the hydantoin lesions site-specifically positioned in 147 bp linear duplexes [20]. Monitoring the glycosylase/lyase activity of the bifunctional DNA glycosylase NEIL1, we have shown that the DNA damage-sensing NER factor XPC reduces the rates of incisions of hydantoin Gh or Sp lesions embedded in double-stranded DNA. Numerical analysis of the kinetic data indicates that both NEIL1 and XPC proteins bind rapidly to Gh or Sp substrates with rate constants close to the diffusion limit for bimolecular association rate constants of other proteins [91,92]. Thus, the preliminary partitioning of binding of NEIL1 and XPC to Gh/SpDNA hydantoin DNA lesions is determined by free diffusion mechanisms. At cellular levels, similar competitive processes between the NER XPC and BER NEIL1 proteins may play a role in the 5–10 times more efficient repair of hydantoin lesions by BER than by NER mechanisms in intact cells [19]. In human mesothelial cells, the nuclear concentrations of NEIL1 are in the range of 250–800 nM [93], while the concentrations of XPC in human fibroblasts were reported to be 140 nM [94].

## 7. Concluding Remarks and Future Outlook

The major pathways of repair of DNA lesions include the base excision repair (BER) mechanism that excises small non-bulky, oxidatively generated DNA lesions, and nucleotide excision repair (NER), a mechanism that removes a large spectrum of mostly bulky DNA adducts generated by UV irradiation or environmental pollutants. A small number of non-bulky, oxidatively generated lesions such as the 5,8-cyclopurine lesions [95,96] are exclusive substrates of NER. However, the oxidatively generated hydantoin lesions (Gh, and Sp), derived from the oxidation of 8-oxoguanine, are substrates of both NER and BER [14,19,20,21]. The interplay between these major repair pathways opened the possibility of comparing the effects of DNA length and topological constraints due the cyclization of DNA, on the relative efficiencies of repair by these two repair pathways. The slower NER efficiencies in the case of linearized forms of the plasmids are correlated with the dissociation of the NER DNA lesion recognition factor XPC from the ends of the linearized plasmid that diminishes the NER efficiency. By contrast, cyclization diminishes the BER yields of the same lesions by ~10–20%. We proposed the hypothesis that these effects can be attributed to the differences in the lesion search mechanisms of BER and NER proteins, that deserve to be investigated in detail. Future studies of the effects of chromatinization of circular instead of linearized plasmid substrates may provide new insights into the poorly understood mechanisms of DNA repair in chromatin contexts that requires the removal of histone core particles by chromatin remodeling factors [86,87,88,89]. The relationship between different pathways of DNA repair could provide a better understanding of the etiology of various human diseases that are associated with oxidatively generated DNA damage and the inflammatory response.

## Figures and Tables

**Figure 1:**
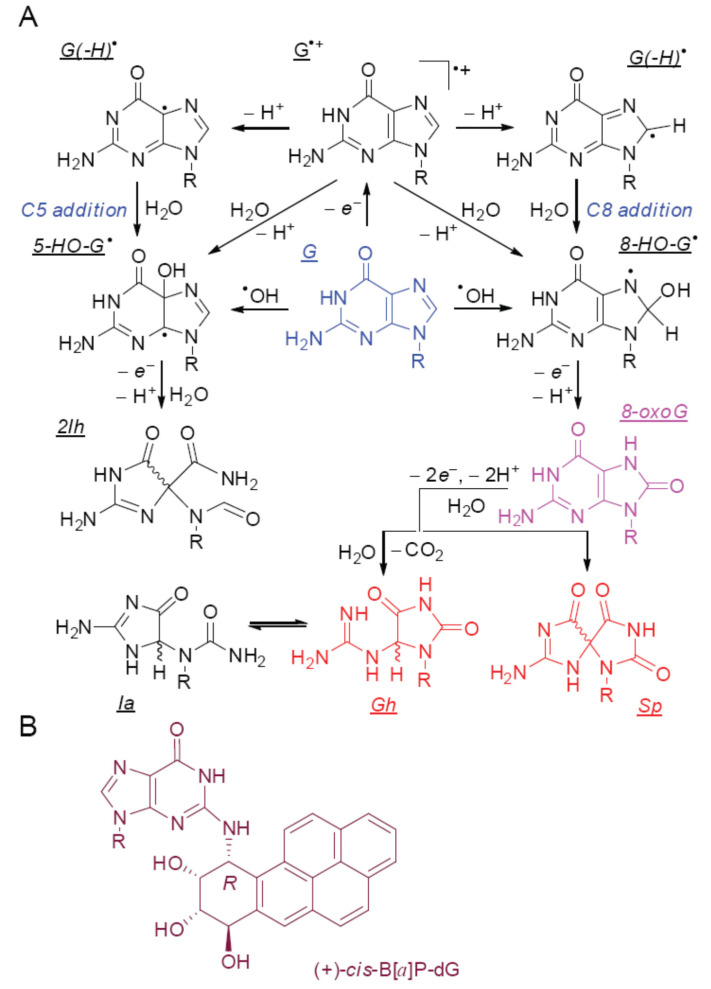
(**A**) Oxidatively generated guanine lesions generated by reactions of strong oxidants or electrophilic free radicals. 8-Oxo-7,8-dehydroguanine (8-oxoG, via C8 addition of H_2_O/^•^OH), and 5-carboxamido-5-formamido-2-iminohydantoin (2Ih, by C5 addition of H_2_O/^•^OH), whereas end products of a four-electron oxidation are spiroiminodihydantoin (Sp) and 5-guanidinohydantoin (Gh). The two-electron oxidation products are substrates of BER only [29,30], while the four-electron oxidation products are substrates of both BER and NER pathways [14,21,31]. (**B**) The bulky guanine DNA adduct 10*R*-(+)-*cis-anti*-B[*a*]PDE-*N*^2^-dG is a well-known substrate of NER only [32,33] and is used for comparing the NER yields of oxidative DNA lesions that are substrates of NER and BER.

## Data Availability

The data that support the findings of this study are available from the corresponding author upon reasonable request.

## Abbreviations

BER	base excision repair;
NER	nucleotide excision repair;
8-oxoG	8-oxo-7,8-dihydroguanine;
2Ih	5-carboxamido-5-formamido-2-iminohydantoin;
Sp	spiroiminodihydantoin;
Gh	5-guanidinohydantoin;
Ia	iminoallantoin;
B[*a*]P-dG	10*R*-(+)-*cis-anti*-B[*a*]PDE-*N*^2^-dG adduct;
G^•+^	guanine radical cation;
G(-H)^•^	guanine neutral radical;
bp	base pair.
